# Genomic features of *Mycoplasma bovis* subtypes currently circulating in France

**DOI:** 10.1186/s12864-022-08818-9

**Published:** 2022-08-19

**Authors:** Chloé Ambroset, Aurélie Peticca, Agnès Tricot, Florence Tardy

**Affiliations:** 1 Université de Lyon, VetAgro Sup, Anses laboratoire de Lyon, UMR Mycoplasmoses Animales, 69280 Marcy l’Etoile, France; 2 Present Address: Université du Mans, Laboratoire Biologie Des Organismes, Stress, Santé, Environnement (BiOSSE), Avenue Olivier Messiaen, 72085 LE MANS Cedex 09, France; 3 Université de Lyon, Anses Laboratoire de Lyon, VetAgro Sup, UMR Mycoplasmoses Animales, 69007 Lyon, France

**Keywords:** *Mycoplasma bovis*, Genome assembly, Subtype, Whole-genome comparison, Mobile genetic elements

## Abstract

**Background:**

*Mycoplasma (M.) bovis* is a major etiological agent of bovine respiratory disease, which is the most economically costly disease of cattle worldwide. Cattle disease surveillance on *M. bovis* is increasingly using gene-based techniques, such as multilocus sequence typing (MLST), or genome-based techniques such as core genome MLST that both require only partial genomic data. However, accurate up-to-date surveillance also demands complete, circular genomes that can be used as reference to track the evolution of the different lineages. Yet, in France, two of the main subtypes currently circulating still have no representing genome in public databases. Here, to address this gap, we provide and compare three new complete *M. bovis* genomes obtained from recent clinical isolates that represent major subtypes circulating in France and Europe.

**Results:**

Genomes were obtained using a hybrid assembly strategy (Illumina and Nanopore) with fine-tuning of settings and inputs used in the Unicycler assembly pipeline, such as size selection of reads and quality trimming of the FASTQ files. The main characteristics and synteny of the genomes were compared. The three genomes mainly differed by their content in terms of mobile genetic elements, i.e. integrative conjugative elements (ICE) and insertion sequences (IS), a feature that impacts their structure. For instance, strain L15527, representing subtype3 (st3), harbours an exceptionally high number of ICEs, which results in a bigger-sized genome than all those previously described and could be associated with the propensity of st3 to gain and fix mutations through chromosomal transfer mechanisms. In contrast, strain F9160, of st1, is very close to the PG45 type strain isolated in 1961 in the USA, and harbours a huge number of IS. These features may be associated with an evolution towards a host-restricted state or in a “closed” host or environment reservoir until a recent re-emergence.

**Conclusions:**

Whole-genome comparison of the three French *M. bovis* subtypes provides valuable resources for future studies combining epidemiology, phylogenetic data, and phylodynamic methods.

**Supplementary Information:**

The online version contains supplementary material available at 10.1186/s12864-022-08818-9.

## Background

The *Mycoplasma* (*M.*) genus gathers more than 200 species of peculiar, fast-evolving bacteria. They are characterized by small wall-free cells and small-sized genomes with a low G + C content (23%–40%), which results in certain low-complexity genetic regions. They also have a specific codon usage bias (with UGA encoding tryptophan). In the tree of life, *Mycoplasma* species are typically located in long branches, suggesting fast-evolving genomes [1]. For instance, *M. gallisepticum* was shown to have the fastest evolutionary rate of all bacteria, with a nucleotide substitution rate of 0.8–1.2 × 10^–5^ per site per year [2].

Comparative genomics is essential to monitor the evolution of bacterial species and the associated epidemiological trends, but comparative genomics studies on mycoplasmas still lag behind most other bacterial pathogens. For instance, the well-known human pathogen responsible for community-acquired pneumonia, *M. pneumoniae*, has only 186 genome assemblies at NCBI [3], while for instance *Streptococcus pneumoniae,* another agent of children pneumonia, has 9048. *M. bovis* holds the record within the genus *Mycoplasma*, with more than 243 genome assembly and annotation reports available at NCBI [3], which reflects rising veterinarian and scientific community concern worldwide around this emerging pathogen [4]. *M. bovis* is responsible for a range of severe infections in cattle, such as pneumonia, mastitis, arthritis and otitis. It is a major causative agent in bovine respiratory disease (BRD), with severe negative impacts on the beef industry and dairy farms worldwide.

The type strain of the species, *M. bovis* PG45^T^, was isolated in 1961 in the USA from the milk of a cow with mastitis, and a laboratory clone was not fully sequenced until 2011, i.e. 50 years later [5]. Fortunately, more recent strains have been sequenced since, and 48 complete genomes are available today in the NCBI database [3]. If we i) focus only on strains from the animal species *Bos taurus* (*n* = 24), i.e. excluding those from bison, ii) remove (‘duplicate’ genomes corresponding to in vitro passages of a same strain, and iii) add two further genomes available but not retrieved by the NCBI browser, we end up with 24 complete genomes, of which only one is from France (Additional file 1), which is a surprising outcome given that France has a long history of *M. bovis* infections [6–12].

Some years ago, our laboratory proposed a simple methodology to infer the population structure of *M. bovis* strains circulating in France based on a single locus sequence typing scheme using 486 bp of the DNA polymerase-encoding gene, *polC* [6]. Three main subtypes were defined: one (st1) described as receding since the year 2000, another (st2) described as dominant, and a third (st3) described as potentially emerging after 2011 [6, 7, 13]. This epidemiological picture was further associated with antimicrobial resistance patterns that differed between the three subtypes. The st2 and st3 isolates were found to be multiresistant to all families of antimicrobials except fluoroquinolones [13, 14], whereas st1 isolates were more susceptible [8, 14]. Furthermore, st3 isolates were shown to more quickly acquire and fix mutations in their quinolone resistance-determining regions (QRDR) in vitro under selective pressure, leading to fluoroquinolone-resistant phenotypes [13]. In contrast, with the same number of in vitro passages, st2 and st1 failed to acquire substitutions in these targets and to evolve towards resistance to fluoroquinolones. Fortunately, a reasoned used of fluoroquinolones has so far limited the apparition of resistant, st3 clinical isolates in France. This subtyping scheme originally developed in France has since been successfully adopted in Spain [15], where it again found a link between subtypes and resistance to fluoroquinolones.

It is important to note that the clonal emergence and dominance of st2 in France (80% of the currently circulating isolates) is not observed in other European countries, where st2 remains rare if present at all [15, 16]. Furthermore, the other two French subtypes have not evolved along the trajectories originally expected. Despite its capacity to fix resistance-associated mutations, st3 has so far been contained to no more than 20% of the *M. bovis* population in France. In contrast, since 2014, st1 isolates have started to be collected again, albeit rarely [7], within the framework of the epidemiological surveillance network Vigimyc [17]. As the capabilities of the different subtypes regarding their evolution toward antibiotic resistance would suggest a dominance of st3, it remains largely unknown why st2 is still dominant and st1 reappears at low prevalence in bovine populations.

Here we set out to conduct an in-depth examination of the genomic features of each of these three ‘French’ *M. bovis* subtypes. For that purpose, we first selected representative strains already enlisted in previous studies, i.e. i) isolates L15762 (st2) and L15527 (st3) used in a study tracking mutation acquisition in QRDR under selective pressure [13], and ii) isolate F9160, which was the first st1 isolate detected in France after the year 2000 [16]. Complete genomes were obtained using the Unicycler pipeline in hybrid assembly mode (Nanopore and Illumina methods) [18]. Comparative genomic analysis showed that the three strains mainly differed by their composition and content in mobile genetic elements (MGEs), i.e. integrative conjugative elements (ICE) and insertion sequences (IS).

## Results

### Complete *M. bovis* genome assemblies required long-read selection and stringent quality trimming

Hybrid de novo genome assemblies were obtained for the three strains using a combination of ONT and Illumina reads, the Unicycler pipeline [18], and different combinations of minimum read length, Nanopore read quality score Q, and mean target coverage (Table 1, Additional file 2). With default Unicycler settings, only strain L15762 was successfully circularized, while 10 contigs and one non-circular contig were generated for strains F9160 and L15527, respectively. A small contig of 5,386 bp was also generated for strains L15762 and L15527 that corresponded to the PhiX174 *E. coli* phage used for quality and calibration assessment of Illumina sequencing and was therefore removed from the final data [19].

**Table 1:** Summary of the main characteristics of the three sequenced *M. bovis* strains

	*M. bovis* strains		
	L15527	L15762	F9160
Isolation date	2011	2012	2014
*polC* st, pubMLST ST	st3, ST21	st2, ST8	st1, ST12
Genome size (bp)	1,187,771 bp	1,006,812 bp	1,026,553 bp
G + C content (%)	29.10%	29.30%	29.20%
ANI vs PG45^T^	98.02%	98.54%	99.62%
Nr. of CDS	1,025	884	925
Nr. of tRNA	35	35	35
Nr. of rRNA (23S, 16S and 5S) sets	2	2	2
Nr. of ICE (vICEBPG45-1 // ICEBPG45-2)	1 // 5	1 // 1	1 // 0
Nr. of IS	80	66	103

The complete genome sequence of *M. bovis* type strain PG45^T^ was used as reference for the calculation of ANI. tRNA and rRNA-encoding genes are counted in the total number of coding sequences (CDS). The pubMLST subtypes were defined using the https://pubmlst.org/organisms/mycoplasma-bovis databasis

In an effort to successfully assemble the three genomes and improve the quality of assembly, we re-ran Unicycler with modified inputs and settings: minimum ONT read length was set at 10,000, 20,000, or 30,000 bp; minimum Q score was set at 11, 13 or 14 (instead of Q7 by default), and the trimming was set to retain reads covering 500 Mb, 300 Mb or 100 Mb (Additional file 2). We obtained a second small contig of 4,937 bp in the genome of strain L15527 that was a chimeric assembly of 4 coding sequences (CDS) already found in the main chromosome (HP, tRNA-leu and 2 ABC transporters) and it was therefore removed from the analysis. The final parameters retained for each genome assembly are summarized in additional file 2, together with the corresponding Busco scores used to evaluate completion of each assembly [20].

All three genomes showed the low G + C content (~ 29%) and other genomic features typical of *M. bovis*, such as two sets of rRNA (23S, 16S and 5S) and 35 tRNA loci (Table 1). The total number of predicted CDS varied from 884 for strain L15762 to 925 for strain F9160 and 1,025 for strain L15527. The three genomes were highly similar with an ANI calculated using PG45^T^ as a reference varying between 98.0% for L15527 to 99.6% for the closest strain F9160 (Table 1). The genome size of strains L15762 and F9160 was coherent with other completely-sequenced *M. bovis* isolates available in databases, in a range of [0.948516–1.165290 Mb] (Additional file 1), but isolate L15527 surprisingly had a bigger genome of 1.187771 Mb.

### ICE composition of the individual genomes explains their differences in genome size

**Fig. 1:**
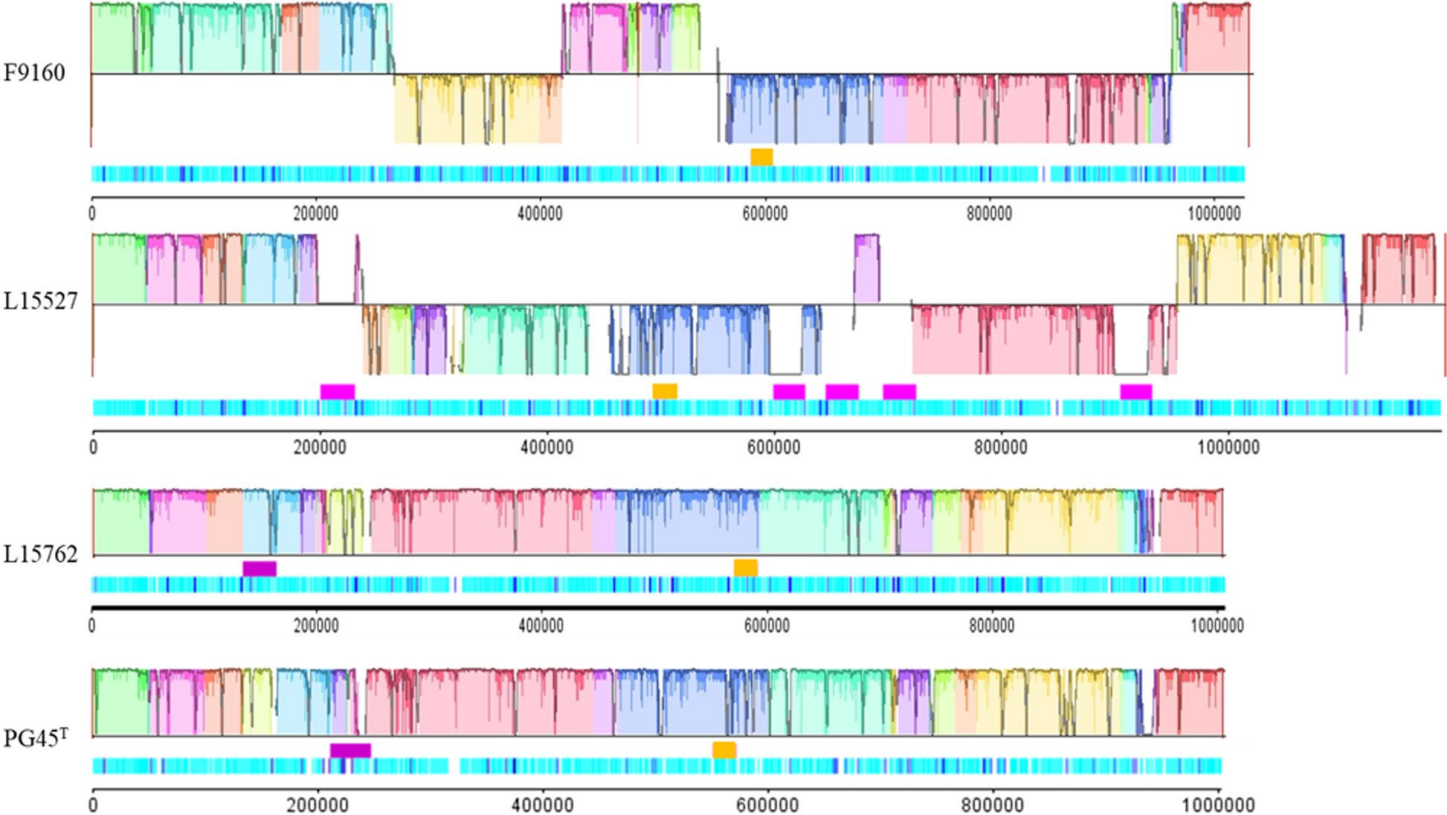
Multiple genome alignment and distribution of mobile genetic elements of the three *M. bovis* genomes obtained in this study and the PG45^T^ reference strain. At the bottom of the Mauve alignments, a linear representation of the genomes (done using DNA plotter) shows CDS in light blue, IS in dark blue bars, and ICEs in orange (vICEB_PG45_-1) or purple (ICEB_PG45_-2) boxes. The alignment process generated a maximum of 16 locally collinear blocks (LCB) representing homologous DNA regions shared between strains without sequence rearrangements. The vertical bars in a LCB denote the conservation level, upward orientation and downward orientation of LCBs relative to the genome, and lines indicate collinear and inverted regions, respectively

The presence of ICE was searched by BLASTn analyses targeting different ORFs of the ICE backbone. Only one copy of vICEB_PG45_-1 was detected in all genomes (Table 1). ICEB_PG45_-2 was absent from F9160 but present in one copy in L15762 and five copies in L15527 (Fig. 1, Table 1). The presence of this exceptionally high number of ICEs explained the atypical genome size of the L15527 isolate. PCR assays were run to further check that this was not the result of assembly errors, and all the amplicons generated had the expected size (Additional file 3). Sanger sequencing results further confirmed the number of ICEB_PG45_-2 and their position in the genome. Note that the ICEB_PG45_-2 copies were highly similar to each other with 99% shared identity over 93% of their total length. Their size varied between 29.4 and 30.2 kbp, with ICEB_PG45_-2_1 being interrupted by an IS30-family IS.

### IS are only a minor factor in the size difference but may influence genome synteny

We also used ISFinder [21] to investigate the presence of IS as another component of the mycoplasma mobilome. Analysis identified 66 IS elements in the L15762 genome, 80 IS in the isolate L15527 genome, and 103 IS elements in the F9160 genome. Compositions in terms of IS families also varied between strains. Strain L15527 had a majority of IS1634-family IS whereas F9160 and L15762 had a majority of IS30-family IS. At a mean size of ~ 1.8 kbp ([1340 bp for ISMbov3–2418 bp for ISMbov5]), the contribution of IS to overall difference in genomic size between strains was limited to 38.5 kbp between F9160 and L15762.

The synteny of the three genomes and PG45^T^ used as reference was compared using Mauve [22]. The structure of the L15762 genome was closer to PG45^T^ than to the F9160 and L15527 genomes, with several locally collinear blocks (LCB) in the same orientation (Fig. 1). There were numerous rearrangements of synteny blocks between the three genomes, suggesting several recombination events. IS, shown as dark blue bars on the bottom charts of Fig. 1, might contribute to these genome rearrangements, as they often flank the inverted coloured blocks. This important rearrangement rate was particularly marked for the F9160 genome, which was highly similar to PG45^T^ with an ANI of 99.6% but with a large number of IS.

### Shared genes and singletons analysis

**Fig. 2:**
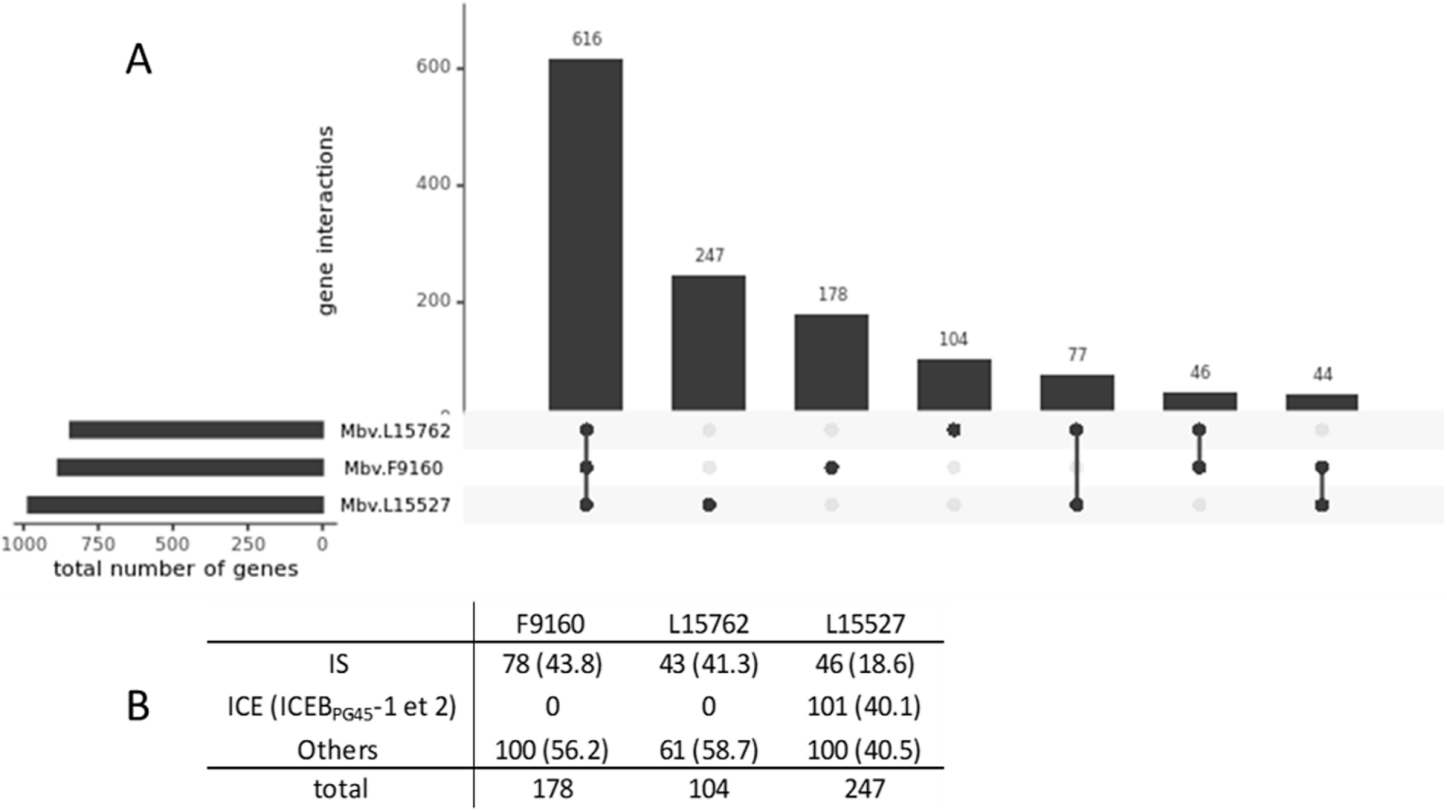
Core-genome and pan- genome representation of the three *M. bovis* strains. **A** Distribution of genes shared or not by the three sequenced *M. bovis* strains (tRNA an rRNA-encoding genes are excluded). Presence of a set of genes in a strain is represented by a black circle. For genes specific to one strain only, there is one black circle only. The horizontal bar chart at the bottom left represents the total number of genes for each strain. **B** Table showing the number of singletons in total or associated with MGEs. Numbers in brackets represent the percentage of each category as a ratio of total number of singletons

The core genome of the three strains is composed of 616 genes, representing 69.7% of L15762 and 60% of L15527 (Fig. 2, A), as expected in a homogeneous species. Strains L15762 and L15527 shared more genes in common (*n* = 77), compared to F9160 with either L15762 (*n* = 46) or L15527 (*n* = 44) (Fig. 2, A). Consistently with its genome size, isolate L15527 had the greatest number of singleton genes (*n* = 247), followed by F9160 (*n* = 178) and L15762 (*n* = 104). Number of singletons was largely driven by duplicated regions and mobile genetic elements (MGE, i.e. IS and ICE) (Fig. 2, B). For instance, strain F9160 harbours a specific 25.5 kbp region flanked by two IS elements that is a complete duplicate of an original 18.5-kbp region with additional duplicated blocks of two CDS, of which one is a transposase (Fig. 1, white LCB, Additional file 4). This genomic island comprises 30 CDS including hypothetical proteins (HP) and IS elements but also duplicated CDS with predicted functions such as nucleotide excision repair and carbohydrate or nucleoside metabolism. Among the singletons of isolate L15527, 37.7% (*n* = 93) were HP and only 2.8% (*n* = 7) were duplicated CDS flanked by IS with known functions. Almost 41% (*n* = 101) of L15527 singletons resulted from duplications of ICEB_PG45_-2 (Fig. 2, B). Among the 104 singletons of L15762, only three had predicted associated functions linked to defence mechanisms in bacteria (methylase modification or DNA helicase) and none of these three CDS resulted from a duplication event.

## Discussion

Three full genome sequences from three *M. bovis* strains isolated from cattle in France were generated using a hybrid assembly approach. Long ONT and short Illumina paired-end reads were combined using the Unicycler pipeline, a method known to optimize bacterial genome assemblies [18, 23, 24]. Despite DNA size selection up to 10 kbp after constructing libraries, we still had to use long read lengths and quality trimming assays to circularize two of the three genomes. Genome announcements rarely report these supplementary steps, suggesting that *M. bovis* genome circularization is fairly straightforward. However, our results highlight that there is no single simple automated process for completing the *M. bovis* genome, largely due to the huge number of IS and ICE it harbours. The low G + C content of mycoplasma genomes and the resulting low-complexity regions also poses challenges for accurate base calling for Nanopore sequencers [25]. Trained base calling using Bonito instead of Guppy offers another promising approach [26], but it will still not solve issues linked to genome structure, notably with repeated varied-length elements such as IS and ICE.

The three genomes studied here are highly similar to each other, with an ANI ranging from 98% to 99.6% using PG45^T^ as reference (Table 1) and a large shared core genome (616 CDS). Their overall features (size, number of CDS, %GC, ribosomal operons, and so on) are comparable to those of previously sequenced genomes (Additional file 1), with the exception of the L15527 genome that had far more CDS and a far bigger size than the other complete genomes. These two features are associated with a huge number of ICE copies. ICEs are self-transmissible MGEs that are considered the most abundant conjugative elements in prokaryotes. In mycoplasmas, ICEs are large modular chromosomal regions of 22 to 37 kbp that integrate at random positions in their host chromosome via an excision-integration process driven by a DDE recombinase (encoded by their CDS22). They comprise 17 to 23 CDS, some of which are involved in their maintenance while the others have unknown functions [27]. Citti et al. [28] recently reviewed their role in horizontal gene transfer (HGT) in mycoplasmas. Here we showed that ICEs disrupt the synteny of the L15527 genomes by introducing lineage-specific sequences in LCBs. They also contribute to 40% of the singletons of the genome (Fig. 2B). vICEB-1_PG45_ (22,271 bp, 17 ORFs in PG45^T^) was defined as vestigial and not self-transmissible by analogy to its counterpart in *M. agalactiae* which was shown to be not horizontally transferred in laboratory conditions [29]. Hence, vICEB-1_PG45_ detection in all three genomes studied here, as well as – at least partially- in all complete genomes available elsewhere (Additional file 1), probably reflects vertical inheritance with potential further genetic erosion leading to pseudogenization or loss of CDS. Wise et al. [5] further suggested that because vICEA is similarly positioned in *M. bovis* as in the highly syntenous *M. agalactiae* PG2 genome, its integration must have occurred in a common ancestor of *M. bovis* and *M. agalactiae* prior to speciation. This is consistent with the very high overall ICE prevalence amongst clinical *M. bovis* strains [30]. In contrast, ICEB_PG45_-2 (37,100 bp, 22 ORFs in PG45^T^), which harboured all the core ICE backbone and might be capable of self-transmission by HGT (by analogy with ICEA_5632_ [31]), was absent from F9160 but detected in one copy in L15762 and in an unexpectedly high number of five copies in L15527. This observation suggests that the F9160 isolate might not have been in contact with strains harbouring ICEB_PG45_-2 or might have lost it in the course of evolution or might be less ‘permeable’ to conjugative elements. Interestingly, of the 24 genomes presented in additional file 1, only 33% had a complete copy of ICEB_PG45_-2. Some strains, such as J279 and J228, also showed remnant ICE-CDS suggesting earlier attack by ICEB_PG45_-2 with subsequent erosion leading to genomic scars only. In strain PG45^T^, ICEB-2 is inserted into an IS element implicated in the inversion of a 483-kb region [5]. It is reasonable to speculate on the fitness cost of such a high number of ICEs (12.5% of the genome in strain L15527) and the potential genetic adaptations to counterbalance it, as is the case for *M. agalactiae* strain 5632 which has three ICEA copies and was shown to be fitter in vitro than the PG2 strain that only has one vICE [32].

IS are other widespread MGEs in mycoplasmas. They are small and are the simplest form of transposable elements, coding only the information that confers their mobility, i.e. the transposase [33]. IS can spread within a genome, causing duplications, deletions and rearrangements, but they can also be transferred between genomes or even mediate the transfer of genetic information coding adaptive traits between genomes [34]. In both cases, their presence and activity will affect genome structure, gene expression and fitness. In *M. bovis* PG45^T^, the IS elements (either full or truncated) are known to comprise about 6.4% of its genome [35] and a mean IS density of 6% has been suggested for the *M. bovis* species [36]. Here, IS density was 11.7%, 8.5% and 8.1% in strains F9160, L15527 and L15762, respectively. These IS density values are far less than the 29% density in *Mycoplasma mycoides* ssp. *mycoides* strain PG1 [37] but are nonetheless higher than in PG45^T^ and most other completely sequenced *M. bovis* strains (Additional file 1, IS copy numbers). Here we further confirmed that IS drive synteny modifications between the three strains through recombination events.

As well as IS copy numbers, the IS types also varied between strains, which is a well-known trait of *M. bovis*. Our genomes were found to contain IS belonging to three different families that all rely on a DDE transposase (IS1634, IS3, IS30). Different IS types were detected within each family, except in IS3 which had ISMbov4 only (Additional file 5). IS1634 was originally described in *M. mycoides* subsp. *mycoides* [38] before also being detected in *M. bovis* [36]. It is highly specific, and its spread between bacterial species or genera is fairly limited (as shown in ISfinder, [39]) compared to IS30. Its profile variation (more specifically ISMbov2 and ISMbov3) has been used for strain typing [40].

From an evolutionary perspective, IS elements can have opposing effects on fitness. A long-term evolutionary course experiment showed that IS activity can promote early adaptation but become detrimental over time [41]. IS were also proposed to contribute to the transition between free-living forms to host-restricted states by causing events with positive within-host fitness effects, such as gene inactivation and chromosomal deletions [42]. However, the evolutionary forces that maintain MGEs, whether IS or ICE, in a genome have yet to be completely unravelled.

It is tempting to associate the different MGE compositions of our strains with their different subtypes and known evolution so far. Apart from being self-transmissible, ICEs also play a crucial role in the unconventional chromosomal transfers (CTs) described in *M. agalactiae* [43] and also experimentally achieved between the two related species *M. agalactiae* and *M. bovis* [44]. CTs are key to adapting to various environments through HGTs that contribute to genome information maintenance, rescue or streamlining. Here the strain chosen as representative of st3 has five ICEB_PG45_-2 copies, suggesting a high capacity for CTs and hence a highly plastic and dynamic genome. This is consistent with hypotheses we posited some years ago about its hypermutability (under selective pressure) and its higher variability compared to st2 [13]. A recent study conducted in Spain using the same subtyping scheme as here confirmed our earlier results, with st3 strains accumulating mutations in their QRDR and hence expressing resistance to fluoroquinolones [15]. The higher variability of st3 compared to st2 (represented here by strain L15762) was also illustrated by synteny comparison between the newly-sequenced strains now available (Additional file 6). With the example of *M. bovis* st3-strain L15527, one can further wonder whether the fitness cost of ICEs multicopies has played a role in the limited spread of st3 in the bovine population or in the important propensity to become rapidly resistant at least in vitro. However, we cannot reasonably extend our hypotheses on the genomic plasticity of L15527 to other st3 strains, as none of the st3 strains that have been completely sequenced to date harbors as many ICE copies as L15527, which thus sets this isolate apart as an extreme case, like strain 5632 for *M. agalactiae* species [45].

F9160 also had an interesting profile. This strain was isolated in 2014, even though st1 had been receding in France and Europe since 2000 [6, 16]. F9160 had an ANI of 99.6% compared to PG45^T^ but with a large 25.5-kbp duplicated region and a vastly higher number of IS (103 versus 54). This IS burst is suggestive of an evolution towards a host-restricted state [42] and is fully coherent with our previous hypothesis of an evolution in a ‘closed’ host or environment reservoir followed by a recent re-emergence [16]. The fact that st1 is again starting to be regularly isolated in France (F. Tardy, personal communication) demands specific targeted monitoring to determine whether this re-emergence is associated with a genomic configuration leading to improved fitness.

Lastly, the st2 strain L15762 has a relatively balanced IS and ICE profile that suggests it has reached a stable configuration, which would explain its fitness and ongoing presence as the predominant circulating subtype in France [7].

In conclusion, this work provides the scientific community with access to three new complete *M. bovis* genomes, which could be helpful for studies potentially combining phylogenetic data and phylodynamic methods, as was recently done for another *Mycoplasma* species that is pathogenic to birds [46]. It also expands the genome-based understanding of the differences between subtypes currently circulating in France or elsewhere in Europe. The proposed genomes nicely illustrate the range of diversity of the *M. bovis* species in terms of MGE composition. Furthermore, our genomic analyses argue for the pertinence of *polC* subtyping or other MLST schemes for disease surveillance purposes. MLST schemes have recently come under criticism as CTs could result in a modified subtype-associated allele without a complete change of the genomic background [47]. However, we believe that the benefit–cost ratio of MLST remains sound, as the technique is easy to handle and relatively inexpensive, and connects up nicely with the other genomic features and evolutionary history of lineages.

## Methods

### Strains, culture conditions and DNA extraction

The three *M. bovis* strains included in this study (Table 1) were collected in the framework of the Vigimyc epidemiology and disease surveillance network [17]. They were grown for 30 h at 37 °C in 5% CO_2_ in PPLO broth supplemented as previously described [48], then seeded onto agar plates. Plates were incubated for 2 to 4 days at 37 °C in 5% CO_2_, and one clone per strain was randomly selected with a wooden toothpick and further sub-cultured in 2 mL of PPLO broth. These steps were repeated three times to ensure a homogenous genetic background of the sequenced clone. Genomic DNA was extracted from 2 × 2 mL, 48 h cultures of the final clones using a commercial kit from Lucigen (Epicentre). Quality of the extracted DNA (OD_260/280_ ≥ 1.8 and an OD_260/230_ ≥ 2) was checked using a Nanodrop spectrophotometer (Thermo Fisher). Concentration of the extracted DNA was checked using a Qubit fluorimeter (DNAds BR kit, Thermo Fisher).

### Preparation of libraries and sequencing

Both the Illumina sequencing (paired-end libraries, MiSeq sequencers) and ONT sequencing (MinION technology) were outsourced (Additional file 2). The ONT sequence libraries were prepared by multiplexing DNA extracts of eight isolates (included the three *M. bovis* strains of this study) per flow cell (R9.4.1) after DNA size selection to enrich DNA fragments sized at > 10 kb. Briefly, for each sample, 4.5 µg of purified DNA was used as input in the end-prep step and then purified with AMpure beads. The barcoding step was performed with a NBD114 kit (Oxford Nanopore Technology, Cambridge, UK), and the barcoded samples were then purified with AMpure beads and pooled in an equimolar ratio based on quantitation and fragment-size estimation. Size selection was performed with ~ 9 µg of this pool using a SRE XL kit (Circulomics) as per the supplier’s recommendations. Three µg of size-selected pool was liganded to an AMII adapter, then purified with L fragment buffer. Sequencing was performed using SQK-LSK109 sequencing reagents and GridION × 5 (GridION release 20.10.06). The Guppy version 5.0.11 base caller was used in super accuracy mode to generate the FASTQ files, which included a quality score Q for each base of each read that is correlated to error rates but slightly differs from the Phred score [25]. FASTQ files with a score > Q7 were demultiplexed using a barcode detection score of 72 and a mid-adapter detection score of 60.

### Quality control and genome assemblies

Raw data (FASTQ files for both short and long reads) were analyzed at the IFB Core cluster [49]. Quality of the Illumina reads was checked using FastQC 0.11.7 [50], and low-quality reads were removed using sickle-trim 1.33 with settings -l 50 -q 25 [51] and Trimmomatic 0.39 [52] to remove Nextera adapters. FastQC was then run a second time to evaluate the quality of the final raw data. Quality of the ONT long reads was checked using Longqc 1.2b [53]. Long reads were then trimmed using Filtlong [54] using Illumina paired-end reads as external reference and with different parameters for Nanopore read quality (Q11 to Q14) and read length (10 kbp to 30 kbp) per strain (Additional file 2). Unicycler 0.4.8 [18, 55] was first used with the default settings, i.e. a minimum read length of 1,000 bp and target bases was selected to retain 500 Mb [18, 23]. Annotation was done using the Prokka 1.14.6 pipeline [56].

Assembly statistics were computed using QUAST 5.0.2 [57] with *M. bovis* PG45^T^ genome (GenBank accession number NC_014760.1) as reference. To evaluate the completeness of the reconstructed genome, assemblies were run through Busco 4.1.4 [58] with the following settings: -m geno –auto-lineage-prok and the mycoplasmatales_odb10 lineage database (174 genes).

### In silico analyses for detection of IS and ICE

The presence of IS was searched using the BLASTn tool in the ISFinder database [21, 39]. For ICE detection, BLASTn searches were run against each genome to search for CDS sequences corresponding to the core ICE backbone as defined by Tardy et al. plus CDS3, 14, 16 and 19 [30]. In detail, CDS1 (GenBank locus tag MBOVPG45_0213) and CDS22 (MBOVPG45_0183) were used to search for ICEB_PG45_-2 and CDS3 (MBOVPG45_0495), CDS5 (MBOVPG45_0494), CDS14 (MBOVPG45_0487), CDS16 (MBOVPG45_0484), CDS17 (MBOVPG45_0483), CDS19 (MBOVPG45_0481) and CDS 22 (MBOVPG45_0479) were used to search for vICEB_PG45_-1.

### PCR amplification and sequencing

The presence of five ICEB_PG45_-2 in the genome of the L15527 isolate was checked by PCR. Six primers were designed, one located in CDS22 of ICEB_PG45_-2 (CDS22_ICEB2_uni: 5’-ACACAAGTGGTAATGCTGAA-3’) and the other five located either upstream or downstream of CDS22 (ICEB2-1_R, 5’-TCCGTATTACTTTTCCTGT-3’; ICEB2-2_F, 5’-GTTTCAAGCTTCAATGCCTT-3’; ICEB2-3_R, 5’-ACAGCTTTTCGAATTTGCTC-3’; ICEB2-4_F, 5’-GCAAGTGTTCAAACAAAAGC-3’; ICEB2-5_F, 5’-TGATAGCTGCATCATGTGAA-3’). The amplicons had an expected size of 655 bp, 686 bp, 718 bp, 702 bp and 1000 bp, respectively. PCR assays were conducted in a final volume of 25 µL that contained 1 µL of template DNA, 0.25 µL of primers at a final concentration of 40 µM, 0.5 µL of dNTP mix at a final concentration of 200 µM and 0.25 µL of GoTaq polymerase (Promega). PCR assays were performed in a Biorad thermocycler with an initial denaturation step of 2 min at 94 °C followed by 30 cycles including one denaturation step at 94 °C for 30 s, primer annealing for 30 s at 56 °C, elongation at 72 °C for 1 min, and a final elongation step at 72 °C for 5 min. Sanger sequencing of the PCR products was outsourced (Genewiz, Germany), and sequence comparison (from de novo assembly and Sanger sequencing) was performed using Geneious Prime®.

### Core-genome and pan-genome analysis with Roary

The core genome and pan-genome of the three strains were calculated using Roary 3.13 [59, 60] with default options and with the.gff files of each genome as input.

### Pairwise comparison of average nucleotide identity

Pairwise comparison of ANI values was done using fastANI v1.3 [61] with default parameters. Strains were first ordered based on ANI values by single-linkage hierarchical clustering.

## Supplementary Information

**Additional file 1: Table S1.** Excel file Addfile_1_TableS1_REV.xlsx

**Additional file 2: Table S2.** Excel file Addfile_2_TableS2_REV.xlsx 

**Additional file 3: Figure S1.** Tiff image AddFile3_SuppFigure1_REV.tif 

**Additional file 4: Figure S2.** Tiff image AddFile4_SuppFigure2_REV.tif

**Additional file 5: Table S3.** Excel Addfile_5_TableS3_REV.xlsx

**Additional file 6: Figure S3.** Tiff image AddFile6_SuppFigure3_REV.tif

## Data Availability

The bioproject associated to this study is PRJNA802351. Nucleotide sequence assemblies were submitted to GenBank and are available under the accession numbers CP092777 (F9160), CP092776 (L15762) and CP092775 (L15527).

## Abbreviations

ANI: Average nucleotide identity
BRD: Bovine respiratory disease
WGS: Whole genome sequencing
Busco: Benchmarking Universal Single-Copy Orthologs
CDS: Coding sequences
CT: Chromosomal transfer
HGT: Horizontal gene transfer
ICE: Integrative conjugative elements
IS: Insertion sequence
MGE: Mobile genetic element
NCBI: National Center for Biotechnology Information
ONT: Oxford Nanopore Technologies
QRDR: Quinolone resistance-determining region
st: Subtype
