# Supplementary figures and images for: Genomic features of Mycoplasma bovis subtypes currently circulating in France
Source: BMC Genomics. 2022 Aug 19;23:603. doi: 10.1186/s12864-022-08818-9 (PMC9392320; doi:10.1186/s12864-022-08818-9)

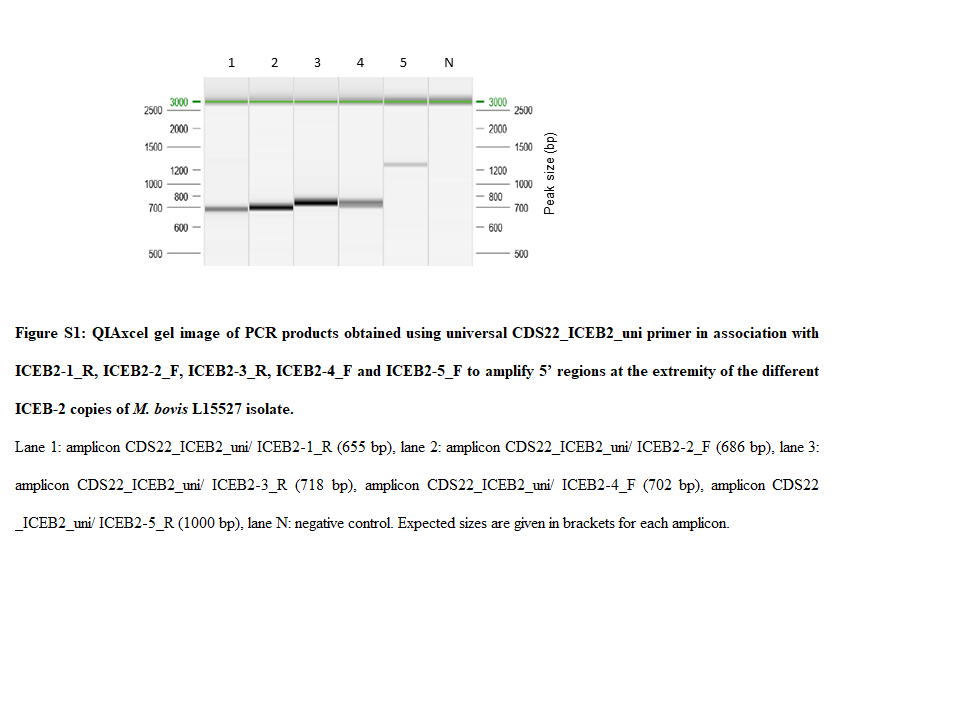

Supplement: Supplementary file 3 — Additional file 3: Figure S1. Tiff image AddFile3_SuppFigure1_REV.tif [file 12864_2022_8818_MOESM3_ESM.tif]

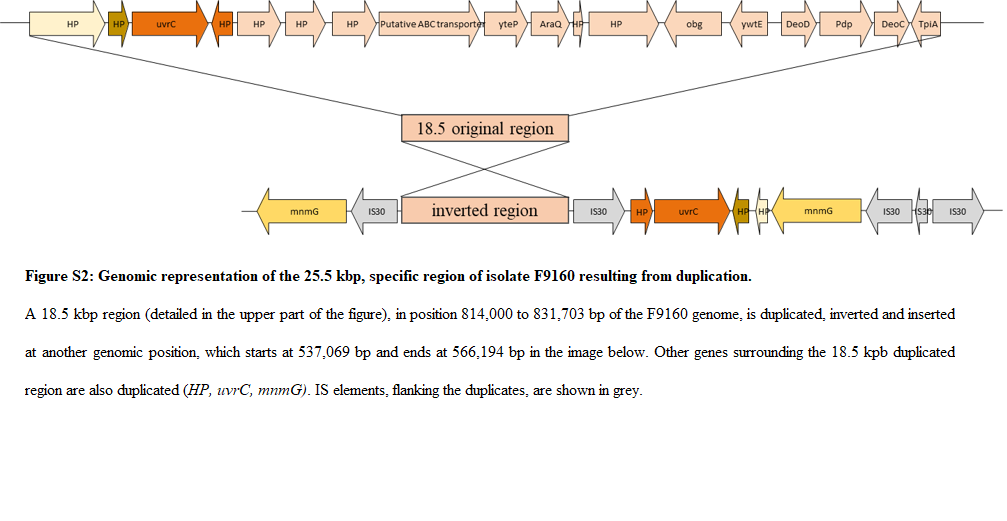

Supplement: Supplementary file 4 — Additional file 4: Figure S2. Tiff image AddFile4_SuppFigure2_REV.tif [file 12864_2022_8818_MOESM4_ESM.tif]

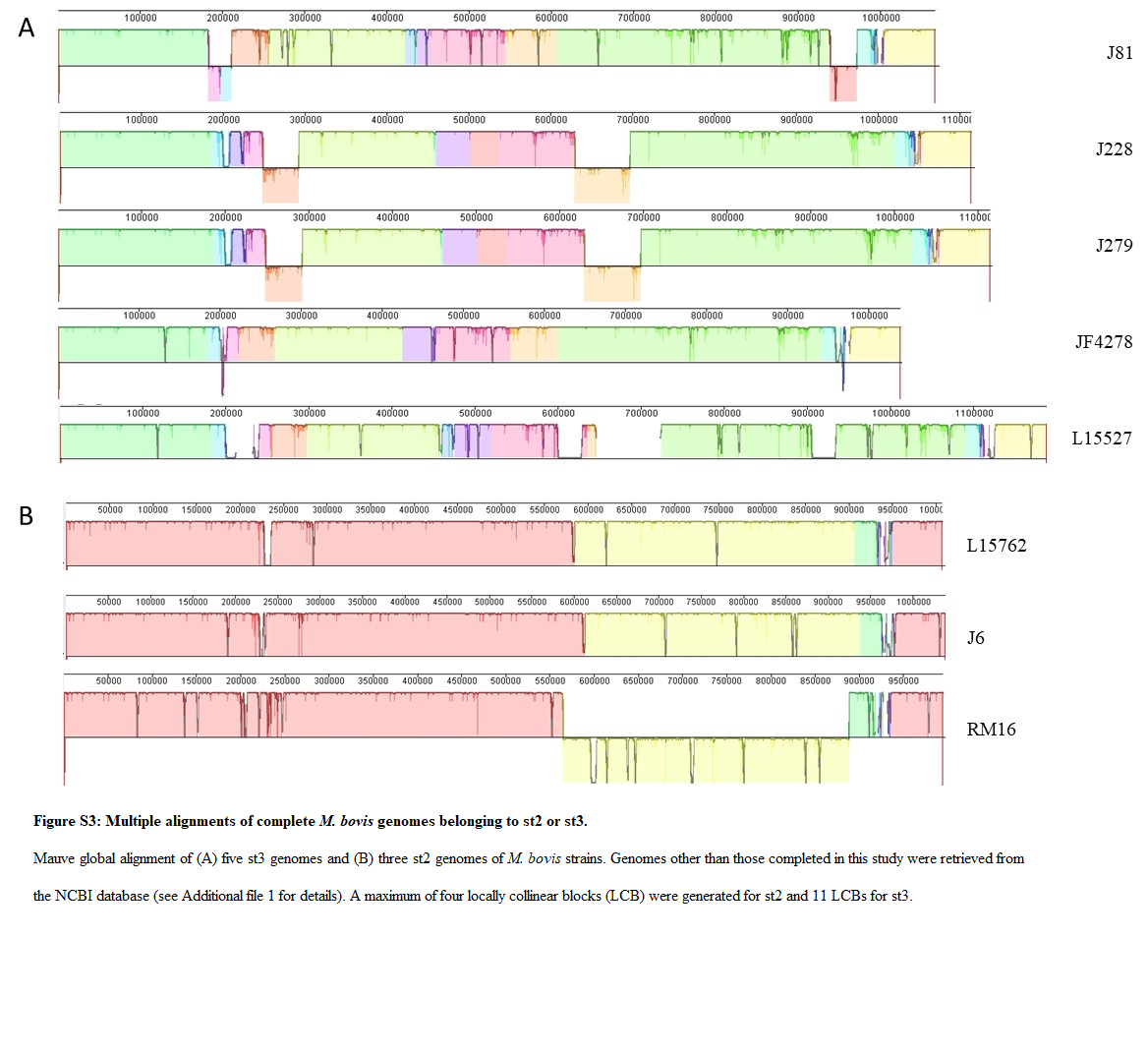

Supplement: Supplementary file 6 — Additional file 6: Figure S3. Tiff image AddFile6_SuppFigure3_REV.tif [file 12864_2022_8818_MOESM6_ESM.tif]
